# Biopsychosocial Insights on Adolescents With Chronic Urticaria: The Role of Eosinophils and Stress Coping Strategies

**DOI:** 10.1002/clt2.70127

**Published:** 2025-12-05

**Authors:** Talat Sarikavak, Sibel Kaplan Sarikavak, Erkan Çakmak, Mehmet Halil Celiksoy

**Affiliations:** ^1^ Department of Psychiatry Istanbul Atlas University Istanbul Türkiye; ^2^ Humanite Medical Center Istanbul Türkiye; ^3^ Department of Pediatric Allergy and Immunology University of Health Sciences Başakşehir Çam and Sakura City Hospital Istanbul Türkiye

**Keywords:** adolescent, children, chronic spontaneous urticaria, chronic urticaria, eosinophils, psychosomatic disorders, severity, stress coping strategies

## Abstract

**Background:**

Chronic spontaneous urticaria (CSU) often lacks a clear etiology. While autoimmune and allergic factors can trigger it, stress and life events also play significant roles. As in other psychosomatic disorders, effective stress coping strategies are key to understanding and managing CSU. This study examined how stress coping strategies relate to biomarkers and disease severity in adolescents with CSU.

**Methods:**

Sixty‐five adolescents aged 12–18 years with CSU and 65 healthy controls were recruited. Both groups completed the Turkish‐adapted Coping Strategies Scale. Sociodemographic data and relevant biological parameters were obtained from the CSU group at admission. Disease severity was assessed using the Urticaria Activity Score (UAS) and the Urticaria Control Test (UCT). Demographic data and coping scores were compared between the patient and control groups, with additional gender‐based comparisons in the CSU group. Regression analysis determined how biological factors and coping strategies explained disease severity.

**Results:**

No significant differences were found in overall sociodemographic data or stress coping abilities between the patients and healthy groups. However, male CSU patients showed stronger coping skills than the healthy cohort (*p* = 0.004). Regression analysis revealed that female gender and higher eosinophil levels were linked to poorer control scores, indicating an interplay between biological factors and psychosocial processes (Std. Bs −0.335 (*p* = 0.016), −0.256 (*p* = 0.006)).

**Conclusion:**

These findings underscore the need for a biopsychosocial approach in adolescents with CSU. Integrating stress management with targeted biological interventions may enhance treatment outcomes and long‐term disease control.

## Graphical abstract

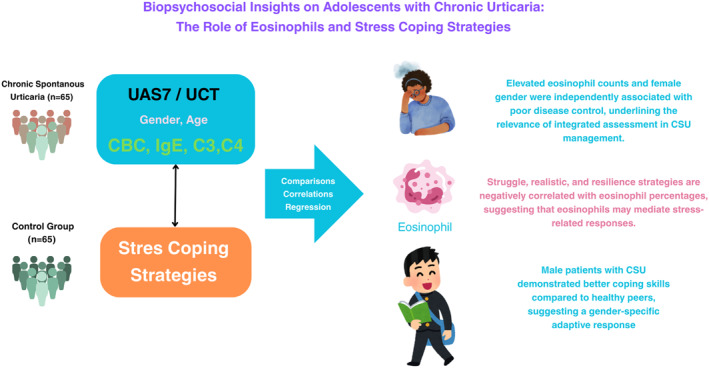

**Abbreviations**
ANA: Anti‐nuclear antibody
CBC: Complete blood count
CIU: Chronic idiopathic urticaria
CSU, CU: Chronic spontaneous urticaria
High‐sensitive CRP: High‐sensitive C‐Reactive Protein
QoL: Quality of life
SCS: Coping Strategies Scale
UAS: Urticaria Activity Score
UCT: Urticaria Control Test

## 1 Introduction

Urticaria is a heterogeneous inflammatory condition characterized by edematous papules or plaques with well‐defined borders, surrounded by an erythematous halo [1]. Chronic urticaria (CU) is defined as the persistence of these symptoms for a duration exceeding 6 weeks. Chronic inducible urticaria is distinguished by the presence of specific, identifiable triggers that precipitate the development of wheals or angioedema, whereas chronic spontaneous urticaria (CSU) is characterized by the absence of a discernible etiology [1, 2, 3]. The clinical spectrum of urticaria encompasses significant variability across its types and subtypes, with the potential for multiple subtypes to coexist within an individual patient [1]. Urticaria has been associated with a significant reduction in quality of life (QoL) and a heightened prevalence of psychiatric comorbidities, most notably anxiety and depression [4, 5, 6, 7, 8, 9]. However, the causal relationship between urticaria and these psychological disorders remains unclear, leaving it uncertain whether anxiety and depression act as precipitating factors or arise as a consequence of the condition [5, 6, 10, 11, 12, 13]. The inability to clearly establish a cause‐and‐effect relationship, or perhaps the possibility that both occur simultaneously, has led us to move beyond these clinical presentations and focus on understanding stress and coping mechanisms. Ultimately, regardless of the clinical presentation of the patient's psychiatric condition, this situation is related to psychological resilience and coping with stress. Directly examining the relationship between these factors and the disease will be crucial in understanding patients and defining areas of intervention.

Coping strategies refer to the cognitive and behavioral efforts people use to manage stress, challenges, or difficult emotions [14]. Although different theories may label coping methods differently, the most widely accepted strategies in the literature are problem‐focused, emotion‐focused, and avoidance coping [15]. Problem‐Based Coping focuses on directly addressing the stressor, Emotion‐Based Coping aims to manage emotional reactions to stressors and Avoidance Coping involves evading stressors [16]. Coping strategies for stress are not innate skills. They are acquired as social and psychological abilities through a developmental process influenced by numerous factors, such as temperament, family attitudes, childhood experiences, and illnesses [15]. In the context of chronic urticaria, the physical symptoms caused by the disease can increase social anxiety and avoidance behaviors [4, 6, 17]. At the same time, through neuroimmunological mechanisms, anxiety and stress can exacerbate and sustain urticaria symptoms [18]. A study conducted on adult patients diagnosed with chronic urticaria found that problem‐focused coping was associated with a reduction in depressive symptoms [19]. Analysis of coping strategies revealed a correlation between emotion‐focused coping and the severity of chronic idiopathic urticaria (CIU) [10, 11].

On the other hand, chronic idiopathic urticaria is a disease with a strong biological component, even though its exact cause cannot always be identified [1]. Various etiological factors play a role in the development of urticaria. Some of these act as primary causes that initiate the condition, while others serve as triggers that exacerbate symptoms. The etiological factors can vary depending on the mechanism of lesion formation, disease duration, and age group [1, 20]. Although many allergic and inflammatory conditions are associated with a peripheral blood eosinophilia, the converse appears to be the case in CSU, with a peripheral blood eosinopenia and basopenia being observed in many patients [21, 22, 23]. Besides eosinophils, and basophils various studies have also found prognostic factors such as neutrophil‐to‐lymphocyte ratio, sedimentation and high‐sensitive CRP to be associated with disease severity scores and response to treatments [22, 24].

This study aims to provide a comprehensive understanding of the interplay between physiological and psychological factors in chronic urticaria management. By examining the role of biological markers alongside stress‐related coping mechanisms, we seek to uncover potential pathways through which these elements interact to affect disease activity and control. Furthermore, this research aspires to contribute to the development of more holistic treatment approaches that address both the biological and emotional dimensions of chronic urticaria.

Specifically, we aimed to gain deeper insights into the experiences of adolescents with chronic urticaria by evaluating their stress coping strategies in relation to disease‐related biological parameters. To achieve this, coping abilities were also compared with those of healthy peers to provide a normative context for interpretation.

We hypothesized that:

1. Adolescents with chronic urticaria would show differences in stress coping strategies compared to healthy controls.
2. Certain coping strategies would correlate with biological markers and disease severity indicators.
3. Both biological and psychosocial factors would contribute to variations in disease activity and control among adolescents with chronic urticaria.

## 2 Methods

### 2.1 Study Population and Laboratory Tests

This study was conducted in the Pediatric Allergy and Immunology Department of Basaksehir Cam and Sakura City Hospital, University of Health Sciences. Patients aged 12–18 years who applied to the outpatient clinic and were diagnosed with chronic urticaria according to established guidelines were included in the study [1]. Those diagnosed with acute urticaria, as well as those with chronic urticaria accompanied by any other chronic or psychiatric condition, were excluded. In addition to patient medical histories, available test results were reviewed, including complete blood count (CBC), blood biochemistry, total IgE levels, C3 and C4 levels, urinalysis, stool examinations for parasites, skin prick tests, ANA, thyroid hormones, anti‐thyroid antibodies, and the autologous serum skin test (ASST). The control group was recruited by announcement from the Pediatric Health and Diseases Outpatient Clinic of Başakşehir Çam and Sakura City Hospital. Controls were recruited clinics for routine exams, with no history of chronic disease. To minimize variations in coping strategies due to developmental differences, we included patients and healthy controls from the same age group with no chronic or psychiatric illnesses.

### 2.2 Scales Used for Evaluating Patients

During follow‐up visits, the Urticaria Control Test, the Chronic Urticaria Activity Score questionnaire, and the validated Turkish version of Coping Strategies Scale (SCS) (48 multiple‐choice questions on a 5‐point scale) were completed by the patients [25]. The SCS comprises 48 items and is divided into three subscales. These subscales evaluate coping strategies related to Struggle (with subscales of Realistic Goal Setting, Resilience, Optimism, Social Interest) Personal Control, and Active Engagement with the Environment. The scale is designed to assess how adolescents manage stress arising from developmental challenges and school‐related factors, such as academic tasks, social interactions, self‐perception, and future concerns. By measuring these coping strategies, SCS provides insights into how young individuals navigate both current and potential stressors in their lives. The *Urticaria Activity Score (UAS)* is used to assess disease activity by evaluating the number of wheals and the severity of itching. Wheals are scored as follows: 0 (none), 1 (< 20 plaques), 2 (20–50 plaques), and 3 (> 50 plaques). Itching severity is rated as 0 (none), 1 (mild), 2 (moderate), and **3** (severe). The *UAS7* score is calculated by summing daily UAS scores over a week, with a maximum possible score of 42 [1]. The *Urticaria Control Test (UCT)* was developed and validated to assess disease control in chronic urticaria over the past 4 weeks. It consists of four multiple‐choice questions, each scored from 0 to 4. The total UCT score is interpreted as follows: **<** 12 (uncontrolled urticaria), 12–15 (well‐controlled), and 16 (completely controlled) [1].

Approval for the study was received from the local ethics committee of Basaksehir Cam and Sakura City Hospital (KAEK/2023.39).

### 2.3 Statistical Analysis

The data in this study were analyzed using SPSS 25 and Jamovi version 2.6.11. To assess the distribution of continuous variables, normality was evaluated using skewness and kurtosis values, with absolute values between 2 and −2, respectively, considered acceptable for normality. Group comparisons were performed using an independent samples *t*‐test for normally distributed continuous variables and the chi‐squared test for categorical variables. Given the large number of analyses, Pearson and Spearmen product‐moment correlation coefficients were calculated to evaluate the strength of relationships between variables.

For categorical variables, proportions and percentages were computed to describe their distribution. Group differences in normally distributed continuous variables were examined using one‐way analysis of variance (ANOVA), while non‐normally distributed variables were analyzed using the Kruskal–Wallis H test. For categorical variables, Pearson's chi‐squared test was applied to identify group differences, and Fisher's exact test was used for categorical data with cell counts less than 5.

Given the exploratory nature of this study and the modest sample size, formal corrections for multiple comparisons (e.g., Bonferroni or FDR) were not applied. Instead, the results are interpreted with caution, emphasizing the consistency and theoretical plausibility of observed associations rather than isolated *p*‐values.

To explore the relationship between urticaria activity and control scores and a set of predictor variables (e.g., sociodemographic, coping strategies, and biomarkers), a stepwise regression analysis was conducted. This approach allowed for the systematic identification of significant predictors while controlling for potential confounding factors. The factors that have collinearity problems excluded.

A prior power analysis performed in Jamovi (version 2.7) indicated that a total of 67 participants would be sufficient to detect medium‐sized effects (partial *η* ^2^ = 0.20, *α* = 0.05, power = 0.80) in a general linear model. Our final sample of 130 adolescents (65 with chronic urticaria and 65 healthy controls) exceeded this requirement. Participants were generally matched by age and sex; however, particular attention was given to ensuring that both groups represented comparable developmental stages, as the primary aim was to evaluate stress coping strategies within similar phases of adolescent psychosocial development.

## 3 Results

### 3.1 Demographics and Clinical Data of Patients

The study involved 65 adolescents with chronic urticaria and 65 healthy children. The mean age of CU patients was 14.86 ± 1.81 years while healthy control group mean age was 14.29 ± 2.73 years. In the chronic urticaria group, 67.7% were female (*n* = 44), while in the control group, 53.8% were female (*n* = 35). There were no gender differences between two groups (*p* = 0.106). The mean age at which symptoms begin in CU patients is 13.169 ± 1.900 years, the mean age at diagnosis is 13.631 ± 1.790 years, and the mean duration of the initial admission for chronic urticaria is 20.241 ± 15.696 months.

Among 65 patients, eosinopenia was present in 15.4% (10 patients), and basopenia was present in 27.7% (18 patients). Among 65 CU patients, eight (12.3%) exhibited sensitivity to inhalant allergens along with concurrent allergic rhinitis. Two (3.1%) had a prolonged urinary tract infection, two (3.1%) had a parasitic infection identified in stool samples, and four (6.2%) tested positive for ANA (antinuclear antibodies). None of the patients had coexisting autoimmune diseases, including autoimmune thyroid diseases, rheumatoid arthritis, insulin‐dependent diabetes mellitus, systemic lupus erythematosus, or autoimmune hepatitis.

All CU patients had chronic spontaneous urticaria, while 22 patients (33.85%) had accompanying symptomatic dermographism, the mean UAS7 score was 23.108 ± 9.309, and the mean UCT score was 8.785 ± 3.286 in CU patients. Patients with chronic spontaneous urticaria exhibited comparable UAS7 and UCT scores regardless of the presence of symptomatic dermographism (*p* = 0.078, *p* = 0.737). Since all patients in our study were using antihistamines, none underwent the autologous serum test. While all patients' symptoms were managed with standard or high‐dose antihistamines, two patients were also receiving omalizumab; however, none were using corticosteroids.

### 3.2 Comparison of Adolescents With CU and Control Group

No differences were observed between adolescents with chronic urticaria and the healthy control group in the total SCS score or its subscales, including SCS Struggle, SCS Realistic, SCS Resilience, SCS Optimism, SCS Social, SCS Personal, and SCS Environment as shown in Table 1 (*p* = 0.275, *p* = 0.192, *p* = 0.419, *p* = 0.392, *p* = 0.165, *p* = 0.114, *p* = 0.678, and *p* = 0.263).

**TABLE 1:** Comparison of adolescents with chronic urticaria and control group.

	Chronic urticaria (*n* = 65)	Healthy children (*n* = 65)	*p* value
Age (years)	14.86 ± 1.83	14.29 ± 2.73	0.165^a^
Gender (female)	67% (*n* = 44)	53% (*n* = 35)	0.106^b^
SCS‐total	162.65 ± 32.13	156.34 ± 33.41	0.275^a^
SCS‐struggle	52.03 ± 17.17	48.08 ± 17.22	0.192^a^
SCS‐realistic	6.71 ± 2.84	6.31 ± 2.78	0.419^a^
SCS‐resilience	27.31 ± 11.09	25.72 ± 9.90	0.392^a^
SCS‐optimism	10.77 ± 5.10	9.49 ± 5.32	0.165^a^
SCS‐social	7.36 ± 2.72	6.55 ± 3.02	0.114^a^
SCS‐personal	51.03 ± 12.36	50.08 ± 13.74	0.678^a^
SCS‐enviroment	57.05 ± 7.85	55.49 ± 7.91	0.263^a^

Abbreviations: SCS, Stress Coping Strategies Scale; SCS‐environment, engagement with external resources; SCS‐optimism, positive expectation; SCS‐personal, internal self‐confidence; SCS‐realistic, pragmatic appraisal; SCS‐resilience, psychological endurance; SCS‐social, social support seeking; SCS‐struggle, active confrontation; SCS‐total, total coping score.

^a^ Independent sample *t* test.

^b^ Ki‐Square test.

### 3.3 Comparison of Male Adolescents With CU and Males of Control Group

Male adolescents with chronic urticaria (*n* = 21) and males of control group (*n* = 30) revealed that the mean age of the chronic urticaria group (14.29 ± 2.08 years) was similar to that of the control group (15.27 ± 1.72 years) (*p* = 0.072). As demonstrated in Table 2, male adolescents with chronic urticaria had significantly higher scores in *SCS Total, SCS Struggle*, SCS Resilience, SCS Optimism, and SCS Personal compared to healthy males (*p* = 0.004, *p* = 0.007, *p* = 0.039, *p* = 0.003, *p* = 0.011). However, no differences were found in SCS Realistic, SCS Social, and SCS Environment (*p* = 0.058, *p* = 0.180, *p* = 0.072).

**TABLE 2:** Comparison of adolescents with chronic urticaria and control group (group comparisons by sex).

	Male chronic urticaria (*n* = 21)	Healthy male (*n* = 30)	*p* value
Age (years)	14.29 ± 2.08	15.27 ± 1.72	0.072
SCS‐total	165.14 ± 29.73	139.30 ± 30.26	**0.004**
SCS‐struggle	51.52 ± 15.44	39.70 ± 14.50	**0.007**
SCS‐realistic	7.19 ± 2.66	5.70 ± 2.72	0.058
SCS‐resilience	26.90 ± 10.28	21.00 ± 9.44	**0.039**
SCS‐optimism	10.71 ± 4.26	7.33 ± 3.45	**0.003**
SCS‐social	6.71 ± 2.80	5.67 ± 2.64	0.180
SCS‐personal	53.33 ± 11.49	44.37 ± 12.31	**0.011**
SCS‐enviroment	57.52 ± 7.09	52.83 ± 8.61	0.072

	Female chronic urticaria (*n* = 44)	Healthy female (*n* = 35)	
Age (years)	15.14 ± 1.65	13.46 ± 3.16	**0.003**
SCS‐total	161.45 ± 33.48	170.94 ± 29.06	0.189
SCS‐struggle	52.27 ± 18.10	55.26 ± 16.25	0.449
SCS‐realistic	6.48 ± 2.93	6.83 ± 2.76	0.589
SCS‐resilience	27.50 ± 11.57	29.77 ± 8.47	0.334
SCS‐optimism	10.80 ± 5.50	11.34 ± 5.96	0.673
SCS‐social	7.67 ± 2.65	7.31 ± 3.14	0.585
SCS‐personal	49.93 ± 12.74	54.97 ± 13.14	0.089
SCS‐enviroment	56.82 ± 8.26	57.77 ± 6.54	0.579

*Note:* *p* < 0.05 is shown in bold.

Abbreviations: SCS, Stress Coping Strategies Scale; SCS‐environment, engagement with external resources; SCS‐optimism, positive expectation; SCS‐personal, internal self‐confidence; SCS‐realistic, pragmatic appraisal; SCS‐resilience, psychological endurance; SCS‐social, social support seeking; SCS‐struggle, active confrontation; SCS‐total, total coping score.

### 3.4 Comparison of Female Adolescents With Chronic Urticaria and Females of Control Group

**FIGURE 1:**
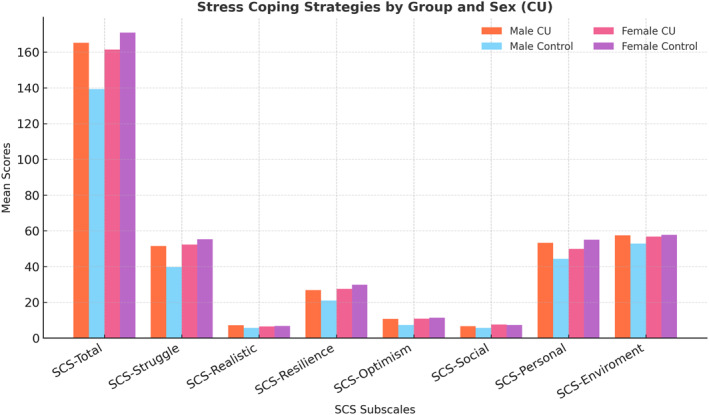
Comparison of stress coping strategy subscale scores by sex and group. Bars represent mean values, and error bars indicate ± 1 SD. Male adolescents with chronic urticaria demonstrated higher total and subscale coping scores compared to healthy males, particularly in the struggle, resilience, optimism, and personal domains. No significant differences were observed between female patients and controls. CSU, chronic spontaneous urticaria; SCS, Stress Coping Strategies Scale.

Female adolescents with chronic urticaria (*n* = 44) and females of control group (*n* = 35) revealed that the chronic urticaria group had a higher mean age (15.14 ± 1.65 years) than the control group (13.46 ± 3.16 years, *p* = 0.003). No differences were observed between female adolescents with chronic urticaria and the female healthy control group in the total SCS score or its subscales, presented in Table 2 and Figure 1, including SCS Struggle, SCS Realistic, SCS Resilience, SCS Optimism, SCS Social, SCS Personal, and SCS Environment (*p* = 0.189, *p* = 0.449, *p* = 0.589, *p* = 0.334, *p* = 0.673, *p* = 0.585, *p* = 0.089, and *p* = 0.579).

### 3.5 Comparison of Male and Female Adolescents With Chronic Urticaria

As shown in Table 3, the mean age of male patients with chronic urticaria (*n* = 21) was 14.28 ± 2.07 years, while female patients (*n* = 44) had similar mean age of 15.13 ± 1.65 years (*p* = 0.079). The age at symptom onset, the mean age at diagnosis, and the duration of symptoms was similar between two groups (*p* = 0.142, *p* = 0.28, *p* = 0.397). Eosinophil and basophil values were similar between males and females (*p* = 0.075, *p* = 0.218).

**TABLE 3:** Comparison of male and female adolescents with chronic urticaria.

	Male chronic urticaria (*n* = 21)	Female chronic urticaria (*n* = 44)	*p* value
Age (years)	14.28 ± 2.07	15.13 ± 1.65	0.079
Age at symptom onset (years)	12.66 ± 2.05	13.91 ± 1.79	0.142
Age at diagnosis (years)	13.28 ± 1.92	13.79 ± 1.71	0.287
Symptom duration (months)	17.61 ± 13.46	21.42 ± 16.62	0.397
Eosinophil count	241.42 ± 195.60	168.63 ± 126.42	0.075
Basophil count	31.42 ± 22.20	25.68 ± 14.69	0.218
UAS7	23.81 ± 10.86	22.77 ± 8.585	0.678
UCT	10.09 ± 2.94	8.15 ± 3.28	**0.025**
SCS‐total	165.14 ± 29.726	161.45 ± 33.48	0.669
SCS‐struggle	51.52 ± 15.44	52.27 ± 18.09	0.871
SCS‐realistic	7.19 ± 2.65	6.47 ± 2.92	0.348
SCS‐resilience	26.90 ± 10.28	27.50 ± 11.57	0.842
SCS‐optimism	10.71 ± 4.25	10.79 ± 5.50	0.953
SCS‐social	6.71 ± 2.79	7.67 ± 2.65	0.186
SCS‐personal	53.33 ± 11.49	49.93 ± 12.73	0.303
SCS‐enviroment	57.52 ± 7.09	56.81 ± 8.25	0.738

*Note:* *p* < 0.05 is shown in bold.

Abbreviations: SCS, Stress Coping Strategies Scale; SCS‐environment, engagement with external resources; SCS‐optimism, positive expectation; SCS‐personal, internal self‐confidence; SCS‐realistic, pragmatic appraisal; SCS‐resilience, psychological endurance; SCS‐social, social support seeking; SCS‐struggle, active confrontation; SCS‐total, total coping score.

Regarding clinical scores, while the UAS7 score was similar between two groups, the UCT score was significantly higher in males (*p* = 0.025). The SCS‐Total scores and other SCS subscale scores including SCS‐Struggle, SCS‐Realistic Goal Setting, SCS‐Resilience, SCS‐Optimism, SCS‐Optimism, SCS‐Personal, and SCS‐Environment, showed no differences between genders (*p* = 0.669, *p* = 0.871, *p* = 0.348, *p* = 0.842, *p* = 0.953, *p* = 0.186, *p* = 0.303).

### 3.6 Correlations and Regression Analysis

Eosinophil percentage (*r* = −0.281, *p* = 0.033), eosinophil count (*r* = −0.272, *p* = 0.039*), platelet count (*r* = −0.326, *p* = 0.013) and sedimentation (*r* = −0.275, *p* = 0.05) were negatively correlated with symptom duration, presented in Table 4. Total IgE and basophil count were found to be correlated with the eosinophil percentage (*r* = 0.357, *p* = 0.004; *r* = 0.283, *p* = 0.023) and eosinophil count (*r* = 0.357, *p* = 0.003; *r* = 0.325, *p* = 0.008).

**TABLE 4:** Correlations and regression for UCT.

	Age (years)	Basofil (*n*)	Total IgE	UAS7	UCT	Symptom duration	Struggle	Realistic	Resiliance
Eosinophil (*n*)		0.325*	0.357*			−0.272*			
Eosinophil (%)		0.283*	0.357*			−0.281**	−0.252*	−0.252*	−0.249*
Basofil (*n*)									
Basofil (%)									
Platelet						−0.326*			
ESR						−0.275*			
UCT				−0.323*					
SCS‐realistic	0.218*					0.276*			

Hierarchical regression model for UCT							
				Overall model test			
Model	*R*	*R* ^2^	Adjusted *R* ^2^	*F*	df1	df2	*p*
**1**	0.283	0.0801	0.0653	5.40	1	62	**0.023**
**2**	0.409	0.1670	0.1397	6.12	2	61	**0.004**
**1–2**	—	—	0.0869	6.36	1	61	**0.014**

				95% confidence interval				
Predictor	Estimate	SE	St. estimate	Lower	Upper	*t*	*p*	VIF
Model 1—Model coefficients—UCT								
Intercept	10.10	0.698		8.70	11.490	14.46	**<** **0.001**	
Gender	−1.98	0.851	−0.283	−3.68	−0.277	−2.32	**0.023**	1.00
Model 2—Model coefficients—UCT								
Intercept	11.65127	0.91044		9.8307	13.47181	12.80	**<** **0.001**	
Gender	−2.46331	0.83915	−0.352	−4.1413	−0.78532	−2.94	**0.005**	0.948
Eosinophil	−0.00645	0.00256	−0.303	−0.0116	−0.00134	−2.52	**0.014**	0.948

*Note:* *p* < 0.05 is shown in bold.

*p < 0.05, **p < 0.001—only significant correlations are written.

Eosinophil percentage was negatively correlated with SCS struggle (*r* = −0.252, *p* = 0.020), SCS realistic (*r* = −0.252, *p* = 0.045) and SCS resilience (*r* = −0.249 *p* = 0.045). ALT (Alanine Aminotransferase) positively correlated with UAS7 (0.269, *p* = 0.032).

SCS Realistic was Positively correlated with Age (*r* = 0.218, *p* = 0.013) and symptom duration (*r* = 0.276, *p* = 0.036). UAS7 is negatively correlated with UCT as expected (*r* = −0.323*, *p* < 0.05).

A stepwise regression analysis identified a significant model for UCT, incorporating gender and eosinophil count as key variables. These factors accounted for 13%, 9% of the variance, with female gender and higher eosinophil count being associated with a decline in UCT scores (Standardized Betas: −0.352, −0.303).

## 4 Discussion

We aimed to investigate the relationships between disease‐associated biomarkers, clinical characteristics, and stress‐coping strategies in adolescents with chronic urticaria in this study. The age group in our study was similar to other studies that included children with chronic urticaria, and consistent with previous research, no gender difference was observed as in other studies [6, 26, 27]. The average UCT score at the first visit of our study patients was 8.7 reflecting poor disease control, which could be attributed to our hospital's status as a tertiary care center. Male gender is linked to better Urticaria Control Test (UCT) scores, aligning with the literature [22]. Although studies in adults have shown worse UAS in females, our study found no such difference [22, 28].

While previous studies linked lower eosinophil counts to higher disease activity, autoimmunity, and poor treatment response, our study found no correlation with UAS7 [22, 29]. However, unlike Kolkhir et al., we observed that lower eosinophil counts were associated with longer disease duration at first admission. Like the literature, both lower eosinophil counts and percentages correlated with lower total IgE levels and basophils [22]. Physical urticarias are linked to a worse prognosis, but in our study, UAS7 and UCT scores were similar in patients with and without dermographism.

In our study, eosinophil count is negatively correlated with the urticaria control score, which may be due to increased stress in CU patients leading to elevated eosinophil counts through hypocortisolism [30]. However, in autoimmune urticaria, it has been associated with eosinopenia. Possible mechanisms include the depletion of blood eosinophils and basophils by recruitment into the skin during active disease and immunologic destruction in the blood [21, 23]. The difference in eosinophil counts and disease severity may be due to variations in the pathophysiological processes between children and adults. There is a need for studies comparing the relationship with eosinophils in the subgroups of chronic urticaria.

In previous studies, eosinopenia was more common in females, which may be due to the inclusion of adult patients in those studies [22]. These findings are consistent with and complement the study by Preis et al., which reported worse outcomes in women with chronic spontaneous urticaria [28].

The comparison revealed no significant difference in stress‐coping strategies between the patient and control groups. However, when the groups were compared based on gender, no difference was observed among females and males with chronic urticaria, whereas males, despite being expected to cope worse, were found to cope with stress more effectively and had better urticaria control scores [31]. The higher stress‐coping scores in the male patient group compared to the controls suggest that men are able to develop coping mechanisms for the disease. However, the inability of female patients to do so may be attributed to upbringing, gender roles, and possibly the greater impact of physical appearance and the skin changes caused by urticaria on girls [31]. In psychosomatic conditions, bodily symptoms can sometimes serve as a means of releasing psychological tension, thereby functioning as a temporary coping mechanism, which sometimes called as “la belle indifference” [32]. However, sociocultural expectations surrounding appearance and self‐presentation may impose additional emotional burdens on female adolescents [32, 33]. These pressures could inhibit the potential “relief” that somatic expression might otherwise provide, leading to a reduced perceived ability to cope with stress despite similar disease burden. This interpretation aligns with prior evidence suggesting that gender norms modulate coping flexibility and emotional regulation in chronic illnesses [9, 32, 33].

The negative correlation between eosinophil count and SCS Struggle, SCS Realistic and SCS Resilience may explain the link between coping with stress and pathophysiology [30]. When interpreted alongside regression results and correlation between eosinophil percentage and symptom duration, it can be suggested that the effect of eosinophil count on disease control is not solely mediated through biological pathways but also through its role in stress coping strategies. The relationship of the MRGPRX2 receptor in mast cells with both eosinophil‐associated proteins and substrates such as CGRP and Substance P released during neurogenic inflammation may represent the cellular‐level reflection of our study findings [34, 35]. On the other hand, the finding that anxiety treatment positively affects the severity and control of urticaria in CU patients serves as further evidence that coping with stress plays a role in both the triggering and perpetuation of the disease [36, 37].

This study offers several key strengths. It provides a comprehensive evaluation of the link between stress coping strategies and chronic urticaria, integrating both psychological and biological perspectives. The use of a stepwise regression model enables the systematic identification of significant predictors of urticaria activity and control, incorporating sociodemographic factors, coping mechanisms, and biomarkers. Additionally, the study's examination of eosinophil counts in relation to stress coping strategies sheds light on the neuroimmunological mechanisms underlying chronic urticaria. Lastly, the findings highlight the clinical significance of addressing anxiety and stress management, advocating for a more holistic approach to improving patient outcomes. Future prospective cohort studies—including the monitoring of psychosocial triggers alongside biological markers—are planned to assess causal pathways and clinical applicability.

However, certain limitations should be acknowledged. The cross‐sectional design restricts the ability to infer causal relationships between stress coping strategies, eosinophil levels, and urticaria activity, necessitating longitudinal research to explore these associations over time. No formal correction for multiple testing was applied; hence, findings should be interpreted as exploratory. Another limitation is the reliance on self‐reported data for stress coping strategies and urticaria control, which may introduce bias due to subjective variability among participants. Moreover, the study does not account for all possible confounding factors, such as medication adherence and environmental triggers. Although the adjusted *R* ^2^ was relatively low, such values are common and acceptable in psychosocial studies, reflecting the multifactorial nature of behavioral and clinical outcomes. Finally, while the sample size is sufficient, it may not fully represent the diversity of chronic urticaria subtypes, particularly in distinguishing autoimmune from non‐autoimmune forms. Future studies with larger and more diverse populations are needed to further validate these findings.

## Author Contributions

**Talat Sarikavak:** conceptualization, methodology, data curation, investigation, formal analysis, writing – original draft. **Sibel Kaplan Sarikavak:** conceptualization, methodology, data curation, investigation, formal analysis, writing – original draft. **Erkan Cakmak:** investigation, validation, resources. **Mehmet Halil Celiksoy:** methodology, investigation, supervision, project administration, writing – review and editing.

## Funding

The authors have nothing to report.

## Conflicts of Interest

The authors declare no conflicts of interest.

## Data Availability

Data available on request due to privacy/ethical restrictions.
